# Increasing research study engagement in minoritized populations: An example from the Black Women Inflammation and Tau Study

**DOI:** 10.1002/alz.14177

**Published:** 2024-08-14

**Authors:** Joy Stradford, Nadine C. Heyworth, Michelle Jackson, Marc Norman, Sarah J. Banks, Erin E. Sundermann, April D. Thames

**Affiliations:** ^1^ SDSU/UCSD Joint Doctoral Program in Clinical Psychology San Diego California USA; ^2^ Department of Neurosciences University of California, San Diego La Jolla California USA; ^3^ Department of Psychiatry University of California, San Diego La Jolla California USA; ^4^ Department of Psychiatry and Biobehavioral Sciences University of California, Los Angeles Los Angeles California USA

**Keywords:** ADRD, African Americans, Alzheimer's disease, Black women, CBPR, CER, community‐based participatory research, community‐engaged research, recruitment

## Abstract

Black women are sorely underrepresented in studies of Alzheimer's disease and related dementias (ADRD) despite higher rates of ADRD diagnoses than in non‐Hispanic White women. There are many reasons for underrepresentation, including medical mistrust, limited access to clinical studies, and restrictive study inclusion criteria. These pervasive barriers to research participation are often not considered during study development and, if eventually thought of tend to be after the fact. Community‐engaged research (CER) approaches are an effective method for reducing participation barriers. This article describes how CER approaches were used to develop the Black Women Inflammation and Tau Study (BWITS), a prospective study to identify biopsychosocial risk factors for ADRD in Black women. Guidelines discussed here for future ADRD research in diverse populations are informed by Community‐Based Participatory Research (CBPR), the National Institute on Minority Health and Health Disparities (NIMHD), and the Patient‐Centered Outcomes Research Institute (PCORI).

**Highlights:**

- Understand the historical tragedies related to medical practices and research designs that may contribute to the underrepresentation of Black Americans in research studies today.
- Highlight community‐engaged research approaches that effectively reduce participation barriers in minoritized groups.
- Review Community‐Based Participatory Research, National Institute of Minority Health and Health Disparities, and the Patient‐Centered Outcomes Research Institute guidelines for conducting research with minoritized communities.
- Describe using the three frameworks to inform the study development protocol for the Black Women Inflammation and Tau Study.
- Conclude by offering study design considerations that we hope can be a helpful starting point for others conducting research with minoritized communities.

## 1 INTRODUCTION

Black Americans are 2–3 times more likely than non‐Hispanic Whites to receive a diagnosis of Alzheimer's disease and related dementias (ADRD).^1^ Considering that women, irrespective of race or ethnicity, are twice as likely to develop dementia than men, Black women are an exceptionally high‐risk group.^2^ A longitudinal study of ADRD risk in women with breast cancer found that the relative risk for ADRD was higher in Black women than in non‐Hispanic White women (odds ratio [OR] = 1.21).^3^ The precise reasons for the increased burden of ADRD in Black women are largely unknown. Some studies suggest that social factors unique to being Black, such as experience with individual and structural racism, discrimination, education, neighborhood disadvantage, and paucity of quality medical care, might account for some of this burden.^4^

Considering the “unknowns” of ADRD risk in Black populations, it is concerning that large‐scale investigations of ADRD and clinical trial studies in general have such low participation rates of Black Americans.^5^ Only 5% of Black Americans participate in clinical observational studies,^6^ and even fewer (1%–3%) participate in ADRD drug trials.^7^ Several reasons may help explain the underrepresentation of Black Americans in clinical research on ADRD, although we review only those reasons that are most commonly cited. This article aims to identify key barriers to ADRD research participation in minoritized communities and to describe an example of how we plan to address these barriers by designing an inclusive and anti‐racist ADRD study, the Black Women Inflammation and Tau Study (BWITS).

## 2 RESEARCH PARTICIPATION BARRIERS

### 2.1 Medical and scientific mistrust

Medical mistrust among African Americans is a complex issue deeply rooted in historical experiences of racism, discrimination, and systemic injustices within the health care system. Medical mistrust significantly impacts participation in clinical trials among Black Americans and other marginalized communities.^8^, ^9^

### 2.2 Exploitive practices

The abusive and exploitative practices toward Black Americans in medical institutions date back to as early as the 17th century when slavery was first documented in the United States.^10^ Owing to the deplorable and inhumane treatment of Black bodies during this time, the infliction of pain on Black bodies was normalized, leading to the rise of myths of Black pain tolerance. Based on pseudoscientific theories of racial inferiority, Black people were misconceived as being able to withstand more pain than other races.^11^, ^12^ Unfortunately, this bias has persisted in today's health care system, as seen by provider bias in pain assessment,^13^ including withholding analgesic treatments.^14^ This is also observed in dementia diagnosis and treatment, as Black Americans often go untreated until later stages of ADRD as compared to non‐Hispanic Whites.^15^, ^16^ The late 19th century marked the start of the Eugenics movement in the United States. The term “Eugenics” was coined by Sir Francis Galton in 1883, with its scientific foundation rooted in social Darwinism.^17^ Eugenics aimed to manually select positive human characteristics to improve future generations, including use of sterilization, segregation, and intelligence quotient (IQ) testing, which targeted Blacks.^18^, ^19^, ^20^ Eugenicists capitalized on standardized IQ testing to fuel racist interpretations about Black inferiority and further promote segregation practices. Stereotypes about Black “feeble‐mindedness” and cognitive inferiority continue to persist in the 21st century. The adverse effects of false stereotypes about Black cognitive inferiority are best demonstrated by the “stereotype threat” experiments by Steele and colleagues.^21^ Furthermore, Thames and colleagues^22^ demonstrated that stereotype threat impacts neuropsychological performance. Consistent with the findings of Steele and Aronson, Thames and colleagues found that Black participants underperformed on testing when the stereotype about cognitive inferiority was made salient.^22^ These findings are particularly relevant to ADRD study designs, considering that cognitive testing is often utilized to assist in diagnostic interpretation.

Other well‐known controversial experiments in the 19th century have influenced medical mistrust today. The unethical and non‐consensual use of biological materials was illustrated by studying Henrietta Lacks’ cell lines.^23^, ^24^ Perhaps the most well‐known study that is a clear violation of medical ethics was the Tuskegee Experiment in Alabama, which aimed to record the natural progression of the sexually transmitted infection, syphilis.^25^ Unethical withholding of medication and treatment of Black male patients with syphilis resulted in hundreds of deaths.^25^

### 2.3 Institutional biases

It is essential to acknowledge that modern‐day medical practices and science continue to harm Black communities. The coronavirus disease 2019 (COVID‐19) pandemic was the most illustrative of these injustices, with higher pandemic‐related deaths and unemployment in the Black community.^26^ Despite the efforts of the National Institutes of Health to provide funding to study COVID‐19, including community engagement, funding is allocated to those who have pre‐established track records of funding, which tend to be White researchers.^27^, ^28^, ^29^ With the increase in funding devoted toward health equity research, a disturbing trend is emerging where researchers with little or no background or training in health equity research, again often White and already well‐funded, are leading grants and publishing papers related to health equity.^30^ This phenomenon illustrates the White racial frame, which describes the predominately White racialized worldview of majority White and White‐oriented decision‐makers in institutional operations.^31^ The White racial frame permeates science, including dementia research, which prioritizes research to document brain health inequities with little interest or investment in developing multi‐systems level interventions that directly address structural racism and racist policies that perpetuate disparities.^32^ Failure to address the root cause of racial differences in dementia rates (e.g., structural racism) runs the risk of misinterpreting scientific findings, and thereby sparking further racist interpretations.

#### 2.3.1 Study design issues

##### Study exclusion

After regulations for human subjects research were implemented, studies began to inadvertently exclude minoritized communities of color from participating in research studies due to their strict study inclusion criteria.^33^ For example, diabetes affects 12.1% of Black American adults^34^; however, individuals with diabetes are often ineligible to participate in ADRD studies, resulting in disproportionate exclusion. High blood pressure is often exclusionary, and 55% of Black Americans have high blood pressure,^35^ therefore unwittingly excluding some of the people who would benefit most from study participation.

Other common exclusionary criteria include comorbid diseases and disorders like cardiovascular disease and obesity, which are more prevalent in the Black community,^36^ and English‐language proficiency requirements that primarily impact minoritized communities of color.^37^ From the recruitment flyers we distribute to the consent forms we provide, many researchers may not realize that we may unintentionally exclude individuals with low reading proficiency and literacy levels.^38^

##### Resource and access limitations

Many assumptions are made regarding the knowledge of and access to study participation. A systematic review conducted by Rivers and colleagues^39^ revealed that low levels of knowledge and awareness about research studies were one of the key issues that impede African American research study recruitment. Studies are typically advertised through various channels within a particular network or geographic area around a medical institution^40^; however, Brooks and colleagues^41^ recommend more widespread direct‐to‐participant advertising to increase study awareness in minoritized groups. Research indicates that greater efforts in recruitment and culturally appropriate outreach will increase African American participation in clinical trials.^42^

Other barriers to study participation include transportation and time.^43^ Having access to reliable transportation to and from study visits and having dispensable time to commit to research or other positive lifestyle activities is a privilege.^44^ Time can be a major obstacle, particularly for individuals with demanding work schedules or conflicting responsibilities, such as work or caregiving duties, making it difficult to allocate time for study participation.^45^ Addressing these barriers through comprehensive and expansive recruitment methods, transportation assistance initiatives, and flexible scheduling options can enhance accessibility and inclusivity in research studies.^43^

Taken together, historical tragedies, individual experiences of unjust treatment in the medical system, and failure to design inclusive studies exacerbate the problem of lack of diversity in clinical research. Thus, establishing or rebuilding trust with minoritized communities and developing inclusive research designs are necessary to foster an all‐embracing and safe research environment and to generate scientific findings applicable and informative to communities of color. The BWITS is one such study that has taken actionable steps toward inclusive study design.

### 2.4 The Black Women Inflammation and Tau Study

**FIGURE 1:**
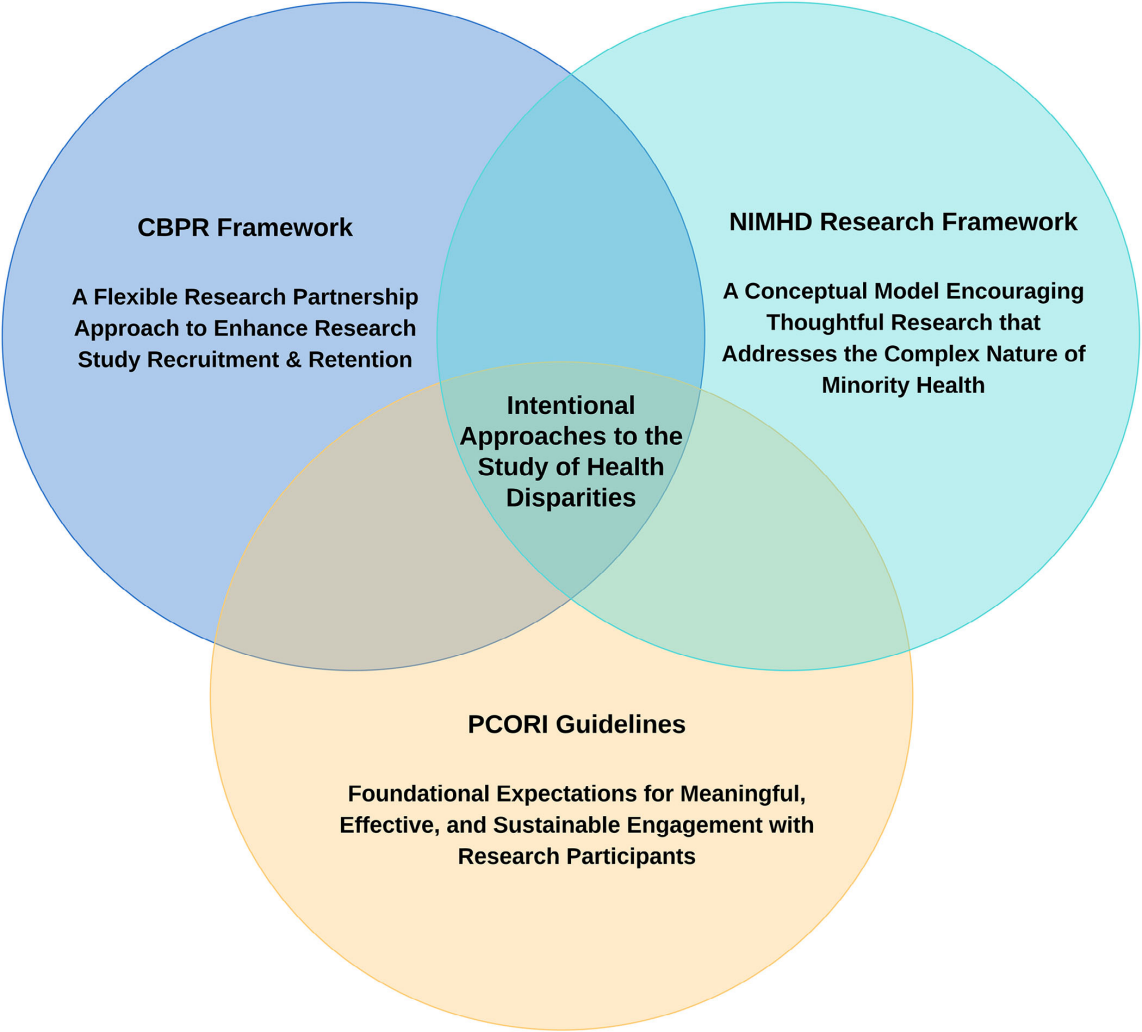
Community‐Based Participatory Research,^44^ National Institute for Minority Health & Health Disparities,^45^ and Patient‐Centered Outcomes Research Institute Frameworks.^46^ Overlapping circles demonstrate independent and synergistic concepts.

The Black Women Inflammation and Tau Study (or BWITS) is a 5‐year prospective observational study recently funded by the National Institute on Aging in 2022. The BWITS aims to determine modifiable biological (neuroinflammation and insulin resistance) and behavioral (physical activity) factors in relation to tau burden (derived from blood sampling) and cognitive function in Black women 60 years of age and older who are at risk for ADRD. BWITS development was informed by the conceptual frameworks of Community‐Based Participatory Research (CBPR), the National Institute of Minority Health and Health Disparities (NIMHD), and the Patient‐Centered Outcomes Research Institute (PCORI) (see Figure 1). BWITS is based out of two study sites in Southern California at the University of California, San Diego, and the University of California, Los Angeles. BWITS aims to recruit 100 Black women across both study sites. Participants will undergo cognitive testing, complete questionnaires that cover demographics, lifestyle factors (diet, sleep, and physical activity), and relevant clinical characteristics (depression and medical history), and undergo venipuncture and an optional sleep assessment at two‐time points—baseline and 24 months later. Study outcomes include cognitive functioning, blood‐derived AD pathology markers, blood‐derived inflammatory markers, objective and subjective physical activity, insulin resistance, diet, social determinants of health (SDoHs), and sleep apnea status and sleep quality/duration (optional).

### 2.5 Frameworks and guidelines that informed BWITS development and study design

The CBPR framework^46^ emphasizes community partnerships and the importance of a multidisciplinary research team. The CBPR framework guided us in selecting members of our research team, community advisory board, and broader partnerships. The NIMHD framework^47^ focuses on individual and societal influences on health outcomes, which guided our team in selecting key study variables. Finally, PCORI engagement guidelines^48^ focus on early and ongoing engagement, which involves ensuring equitable distribution of resources, capacity building, shared decision‐making, and maintaining checks and balances through ongoing review and assessment of partner engagement. Below, we describe specific examples of how these frameworks were implemented in BWITS.

### 2.6 BWITS implementation of CBPR, NIMHD, and PCORI frameworks

#### 2.6.1 Identifying the research team

One of the steps of CBPR is selecting the community (e.g., population of interest), stakeholders from the community, and research partners to help develop the research questions and execute the research design. Given the problematic rates of ADRD in Black women and the underrepresentation of Black women in ADRD research, it was important to select Black women as our population of focus. Although it was important for our research partners to know ADRD risk and lifestyle factors, it was even more important that our research partners had direct experience working with communities of color, particularly Black communities. Furthermore, we incorporated a community advisory board ([CAB] of which we later discuss these steps) comprising active community members. Researchers and community partners support one another, acknowledging that every team member is valuable.

#### 2.6.2 Creation of the community advisory board

Our community stakeholders and members comprise the BWITS CAB, which was formed through a combination of personal connections, professional collaborations, and newly established relationships within the community. The Board consists of seven Black women in Southern California who hold various roles as medical professionals, educators, business owners, caregivers, religious leaders, and community members with the shared lived experience of being Black women themselves. Together, the research partners and CAB draw upon their varied expertise and experiences regarding input on research study development and design. A CAB is pivotal in guiding study design by offering insights and perspectives on developing and implementing research protocols. One of the six PCORI foundational principles for developing a CAB focuses on diversity and representation within its members. The BWITS CAB reflects the diversity of the Southern California Black community. Another foundational principle contributing to thoughtful study development is ensuring early and ongoing engagement with community partners. BWITS has engaged with a CAB since the study's inception, which has improved the study design, ensuring cultural humility in implementing protocols and mitigating the potential for unintended harm or offense. Finally, the community reciprocity tier of the CBPR framework is of major importance. Through forming a CAB, BWITS has received valuable feedback and perspectives from the community that may not be apparent to researchers, and in turn, our team has provided community partners with education about the research process and perspectives of working within the guidelines of the institutional review board and funding agency. This process has ensured that the research is culturally sensitive, inclusive, and equitable.

#### 2.6.3 Ongoing review and assessment of engagement

PCORI promotes ongoing review and assessment of engagement with study partners. BWITS development progress and partnership engagement are monitored continually, and the team actively identifies what is working well and what can be improved based on collective team feedback. Although engagement can fluctuate at different times of the year, our team has brainstormed ways to keep partners engaged, including routine meetings and organizing events that celebrate partners' contributions. PCORI highlights the importance of establishing dedicated funds for increased engagement and partner compensation. BWITS consulted with our CAB regarding both the CAB and broader community partners' compensations to ensure that it was fair and reflective of their time commitment. The CAB has committed to providing feedback throughout the duration of the study about ways to maintain engagement among our broader community partners.

#### 2.6.4 Critical analysis of the protocol

The reducing barriers tier of the CBPR framework and the PCORI guidelines promote the critical analysis of the study protocol with input from community partners. The BWITS team collaborated to create a less‐invasive study protocol than other ADRD studies. For example, some ADRD studies include magnetic resonance imaging (MRI), positron emission tomography (PET) brain scans, and lumbar punctures in their research protocol to examine the pathophysiology of ADRD. The BWITS team focused on blood‐based biomarkers rather than invasive procedures (e.g., brain imaging, lumbar punctures).

Our CAB members have played a major role in decision‐making throughout all project phases. For example, our initial inclusion criteria included participants younger than 65 years of age, and our initial exclusion criteria included diabetes diagnoses. However, our CAB educated the team about the importance of lowering the age requirement and the prevalence of diabetes in the Black population. Therefore, we changed our criteria to include participants 60 years of age or older and those diagnosed with diabetes. This reduced our initial screen fails by 20%. The CAB also emphasized the importance of being transparent about how the study's data was going to be used and shared. Thus, we clarified this in the informed consent form by providing data‐sharing options to elect into or opt out of. These are just a few examples illustrating the benefit of reviewing the study protocol with input from community partners.

#### 2.6.5 Selection of culturally appropriate measures

The NIMHD framework specifies domains of influence over the life course that may impact individual health, including the sociocultural environment. Therefore, when working with diverse and hard‐to‐reach populations, ensuring that the selected measures do not offend or harm the population you wish to serve is imperative. The BWITS team worked together to select culturally appropriate measures for our test battery. After the test selection, the CAB carefully combed through each cognitive test and questionnaire to provide their opinions about the cultural sensitivity of the selected measures. As a result, we changed the format of administration of questionnaires that are highly sensitive (e.g., asking about early life adversity) to more of an interview style between the psychometrist and the participant rather than handing the questionnaire to the participant to complete on their own. This collaborative approach of careful selection and cultural sensitivity assessment is imperative to ensure minimal risk of harm when working with minoritized groups.

#### 2.6.6 Selection of social determinant of health factors

The individual health domain of influence from the NIMHD framework highlights biological vulnerabilities and mechanisms. By understanding interactions between biological mechanisms and social factors, we can better address health disparities in minoritized communities instead of making incendiary statements about biological inferiorities or “vulnerabilities.” BWITS utilizes blood‐based biomarkers to examine inflammation, tau deposition, and insulin resistance, all of which are associated with dementia outcomes and are disproportionately elevated in Black persons. NIMHD also recognizes the contribution of individual behaviors and structural factors to overall health. BWITS will examine lifestyle factors such as sleep and engagement in cognitive and physical activities that may influence health outcomes.

As highlighted by the NIMHD framework, the onus of health outcomes does not rest solely on the individual. Instead, it is important to acknowledge how one's environment is often driven by SDoH factors. SDoHs are conditions in the environment in which an individual is born, lives, works, plays, and worships that impact a wide range of health, functioning as week as quality of life outcomes and risks.^49^ To address certain aspects of SDoHs, the BWITS will utilize the area deprivation index (ADI) assessed at two timepoints (childhood 0–18 vs current), which ranks neighborhoods by socioeconomic disadvantage based on public data about employment rates, income, education, and housing quality. We will also collect additional SDoH data such as neighborhood safety and green space, medical care accessibility, and perceived discrimination throughout the lifespan. We considered SDoHs in the design of BWITS and made modifications such as having study visits occur in convenient locations within predominantly Black San Diego and Los Angeles communities instead of requiring participants to commute to university research facilities. We believe this will help to address problems related to limited access to reliable transportation. We also considered the financial burden of transportation, which may persist regardless of the study location; therefore, participants will be offered free transportation to and from visits.

#### 2.6.7 Partnering with the broader community to increase access to study participation

The community partnerships tier of the CBPR framework outlines the importance of collaborative science with partners who understand and have shared lived experiences with the communities. Similarly, the PCORI guideline of building the capacity to work as a team expands this point. Thus far, our BWITS team has partnered with churches and retirement centers in predominantly Black San Diego communities. We have also formed partnerships with various Black professional organizations and associations in Los Angeles. This allows further community input into the BWITS study design and procedures and an opportunity for participants to learn how they can participate in ADRD clinical research. Through these efforts alone we have engaged ≈150 Black women who have formally expressed interest in participation. Furthermore, it is crucial to collaborate not only with community organizations but also with individuals actively involved in those organizations who can speak to the credibility of the study. These individuals can help the study gain respect within the community and enhance the impact that the research can have on the community.

#### 2.6.8 Dissemination of results

The Health Care System domain of influence from the NIMHD framework underscores the importance of thoughtful interactions with participants. Discriminatory practices by research study staff^50^ and assumptions about health literacy^51^ can serve as hurdles to study recruitment and retention. BWITS strives to address this by disseminating digestible results to participants. BWITS has created a health dashboard that provides participants with information about their own personal health derived from BWITS measures, including fasting glucose, insulin, HgA1c levels, glomerular filtration rate, blood pressure, mental status examination, and sleep study information. Although the BWITS research team is unable to advise and counsel participants on how to directly proceed with the information from their health dashboard, a BWITS research staff member will review the information with each participant and provide optional resources for physicians and medical centers where participants can elect to pursue clinical follow up regarding their results. The BWITS team will offer to help participants form a list of relevant questions to ask a provider if additional follow‐up is needed. In addition, BWITS has been providing and will continue to provide brain health awareness and outreach within Southern California communities to promote increased awareness of ADRD and learn from the community some of the continued barriers to research participation.

## 3 CONCLUSION

In sum, although efforts and successes have been made to increase diversity representation in ADRD research, problems still exist with retention, engagement, and a true understanding of minoritized communities' traumas that may perpetuate barriers. The BWITS study is one example of how to guide an intentional and thoughtful study design with established frameworks from CBPR, NIMHD, and PCORI. Nevertheless, there are limitations to acknowledge with respect to BWITS. First, our CAB was developed based on recommendations from research team members who already had established relationships with the members. It is possible that the process would differ had the members of the CAB not already had existing relationships with the research team. The two study sites of BWITS (Los Angeles and San Diego) are within Southern California. There may be cultural and life experiential differences that are unique to living in Southern California that may not generalize to Black women living in other parts of the United States. With this said, we believe that the general intention to promote inclusivity across ADRD studies has high potential to close the racial and gender gap in ADRD knowledge. Incorporating frameworks from CBPR, NIMHD, and PCORI has improved our study focused on ADRD risk in Black women. Below are eight steps to consider when designing ADRD research that aims to be inclusive of diverse communities.

## CONFLICT OF INTEREST STATEMENT

The authors declare that the article was written in the absence of any commercial or financial relationships that could be construed as a potential conflict of interest. Author disclosures are available in the supporting information.

## Supporting information


Supporting Information
